# Effect of Aging on Homeostasis in the Soft Tissue of the Periodontium: A Narrative Review

**DOI:** 10.3390/jpm11010058

**Published:** 2021-01-18

**Authors:** Yu Gyung Kim, Sang Min Lee, Sungeun Bae, Taejun Park, Hyeonjin Kim, Yujeong Jang, Keonwoo Moon, Hyungmin Kim, Kwangmin Lee, Joonyoung Park, Jin-Seok Byun, Do-Yeon Kim

**Affiliations:** 1 Department of Pharmacology, School of Dentistry, Kyungpook National University, Daegu 41940, Korea; cosmos0468@naver.com (Y.G.K.); leeyang2324@naver.com (S.M.L.); em5161@naver.com (S.B.); parktj@gmail.com (T.P.); twdt92@gmail.com (H.K.); yjeong07@naver.com (Y.J.); moons2317@naver.com (K.M.); hyuongmin@naver.com (H.K.); mgkd200@naver.com (K.L.); junfriend777@hanmail.net (J.P.); 2 Department of Oral Medicine, School of Dentistry, Kyungpook National University, Daegu 41940, Korea; 3 Department of Pharmacology, School of Dentistry, Brain Science and Engineering Institute, Kyungpook National University, Daegu 41940, Korea

**Keywords:** aging, periodontium, gingiva, periodontal ligament, MMP

## Abstract

Aging is characterized by a progressive decline or loss of physiological functions, leading to increased susceptibility to disease or death. Several aging hallmarks, including genomic instability, cellular senescence, and mitochondrial dysfunction, have been suggested, which often lead to the numerous aging disorders. The periodontium, a complex structure surrounding and supporting the teeth, is composed of the gingiva, periodontal ligament, cementum, and alveolar bone. Supportive and protective roles of the periodontium are very critical to sustain life, but the periodontium undergoes morphological and physiological changes with age. In this review, we summarize the current knowledge of molecular and cellular physiological changes in the periodontium, by focusing on soft tissues including gingiva and periodontal ligament.

## 1. Introduction

Aging is defined as a progressive decline or loss of intrinsic physiological functions, leading to a decrease in reproductive and survival rates [1]. Although death is inevitable to human, individuals have craved a way of delaying the aging process and extending the lifespan. In this regard, aging research has identified critical risk factors and treatment strategies for major age-related pathologies including cardiovascular diseases, neurodegenerative disorders, cancers, and other chronic diseases [2]. In addition, recent studies suggest several promising interventions, such as regulating nutrient sensing, controlling cellular senescence, and balancing the gut microbiome [3]. 

Indeed, some pharmacological approaches were proven to slow down age-dependent functional decline. For example, rapamycin extended the median and maximal lifespan of mice when fed beginning at 20 months of age [4]. Rapamycin seems to extend healthspan as well as lifespan through reversing age-related vascular dysfunction and oxidative stress [5], rejuvenating oral health [6], and ameliorating age-dependent cognitive deficits [7]. Interestingly, recent clinical study showed that topical rapamycin treatment efficiently suppressed human skin aging [8]. Resveratrol, a polyphenol found in red wine, has been also proven to prolong lifespan of model organisms including yeast, nematode worms, fruit flies, and rodents [9,10,11]. Resveratrol additionally showed the protective effects in several mouse models of age-dependent neurodegenerative disorders [12,13]. Recently, resveratrol was reported to enhance cognitive and cerebrovascular functions in postmenopausal women [14]. Metformin, the first-line medication prescribed for type 2 diabetes, was also found to significantly prolong lifespan in worms by up to 36% [15,16]. However, whether these pharmacological interventions extend the lifespan and/or healthspan in humans needs to be validated further.

Aging research proposed critical aging hallmarks that describe common denominators among species, including genomic instability, cellular senescence, and mitochondrial dysfunction [2]. During aging, genetic damages are accumulated and gene expression patterns are continuously changed [17]. These alterations are mediated by extracellular chemical/biological stimuli, as well as by intracellular replicative/oxidative stress. Genetic disintegrity and abnormal gene expression changes often lead to numerous aging disorders [18]. Aging-inducing factors also trigger cellular senescence which can be dependent on or independent of telomere shortening. Cellular senescence refers to the irreversible loss of cell growth capacity, which commonly accompanies peculiar phenotypic alterations. Of note, frequencies of senescent cells are dramatically increased in several tissues, when comparing young and old mice. For example, approximately 8% of senescent cells were observed in young mice liver but the percentage of senescent hepatocytes are increased to 17% in old mice. Additionally, in the spleen, young mice have only 7% of senescent lymphocytes but aged mice have 25% of senescent lymphocytes [19]. Accumulation of senescent cells is also observed in humans in several tissues [20]. When the cellular senescence process is hyperactivated, it leads to downregulation of tissue function and repair, upregulation of inflammatory responses, reduction of cell renewal, and exhaustion of stem cells. Aging also affects the efficiency of the respiratory chain in the mitochondria. Furthermore, mitochondrial dysregulation can expedite aging [21]. Mitochondrial decline can affect apoptotic signaling and upregulated unwanted inflammatory reactions [22]. In addition, inefficient biogenesis of mitochondria with age causes disruption in energy metabolism systemically.

The periodontium, a complex structure surrounding and supporting the teeth, is composed of the gingiva, periodontal ligament, cementum, and alveolar bone [23]. The primary roles of the periodontium are to support attachment for the tooth to the bone of the jaw and help the tooth to endure the stress of mastication. In addition, the periodontium guards the underlying structure against pathogenic oral microflora and protects the blood vessels and nerves from injury. Dynamic remodeling in the periodontium is essential for orthodontic tooth movement, improved occlusion, and decreased tooth wear [24,25]. As a functional biological system, the periodontium undergoes morphological and physiological changes with age. In this review, we summarize the current knowledge of molecular and cellular physiological changes in the periodontium, by focusing on soft tissues including gingiva and periodontal ligament.

## 2. Anatomical and Physiological Homeostasis in the Gingiva

The gingiva is located on the external surface of the periodontium and covers alveolar bone and tooth root. The gingiva is composed of an outer epithelial layer and an underlying connective tissue, called lamina propria, and the epithelium is histologically subdivided into at least three sections (the oral epithelium, the sulcular epithelium, and the junctional epithelium). While the gingival epithelium consists of several cell types, including keratinocytes, melanocytes, Langerhans cells, and Merkel cells, the lamina propria is mostly filled with collagen fibers, vessels, nerves, and matrix [26]. Compared to the epithelial layer, relatively small quantity of cells, such as fibroblasts and innate immune cells, reside in the connective tissue.

The oral epithelium is a keratinized oral mucosa and it is the thickest (0.2–0.3 mm in thickness on average) layer among the gingival epithelium. The sulcular epithelium is a thin, non-keratinized (but often parakeratinized situationally) epithelial lining of the gingival sulcus. The oral and sulcular epithelium primarily perform protective and defensive roles. However, because the sulcular epithelium may act as a semi-permeable membrane through which pathogenic bacterial byproduct penetrate, it can be involved in the detection of bacterial assault and initiation of immune responses. In contrast, the junctional epithelium, non-keratinized cell layer adhering to the tooth surface, serves multiple functions. The junctional epithelium mediates the epithelial attachment to the tooth surface and basement membrane facing the connective tissue through hemidesmosomes and the extracellular matrix [27]. The junctional epithelium also serves as the front-line of defense system against injurious bacterial infection by expressing anti-microbial factors such as secretory leukocyte protease inhibitor (Slpi) [28] and S100A9 [29]. In addition, the junctional epithelium cells constitutively express chemokines and cytokines including keratinocyte-derived chemokine (KC/CXCL1) and macrophage inflammatory protein-2 (MIP-2/CXCL2), as a result, polymorphonuclear leukocytes are heavily infiltrated into the junctional epithelium, which provides maintenance of physiological homeostasis as well as anti-pathogenic defense [30]. Given that the junctional epithelium shows high turnover rate, it can also contribute to tissue regeneration and dental plaque suppression [31].

The gingival connective tissue performs critical functions in the protection of cement root and alveolar bone [26]. The connective tissue also shows rapid turnover, it has a marked capacity of healing and regeneration [32]. In gingival connective tissue, fibroblasts perform central roles in the development, homeostatic maintenance, and repair. Fibroblasts constitute approximately two-thirds of cell population in the gingival connective tissue, and synthesize/secrete components of the extracellular matrix such as collagen, elastin, and glycoproteins. Fibroblasts induce proliferation of connective tissue cells by expressing platelet-derived growth factor (PDGF) [33], and following injury to tissues, fibroblasts migrate to damaged sites and mediate tissue repair/regeneration, in cooperation with inflammatory cells [34].

**Figure 1:**
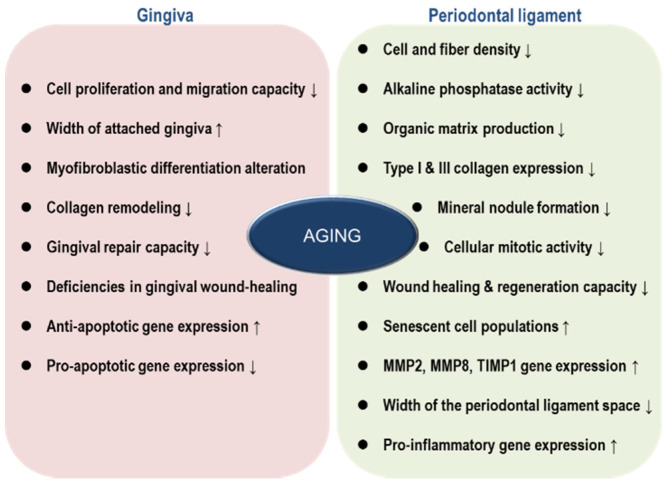
Physiological and biochemical changes in gingiva and periodontal ligament during aging.

Aging seems to negatively affect homeostatic regulation in gingiva (Figure 1). Mounting evidence demonstrated that the width of attached gingiva increases with age, mirroring the riskiness of disease incidence on the periodontium [35,36]. Gingival recession caused by accumulated mechanical trauma or gingivitis could induce the loss of attachment, in an age-dependent manner [37]. Cáceres et al. compared several physiological aspects of young and aged gingiva, with primary cultures of gingival fibroblasts and rat models. Importantly, gingival fibroblasts from aged individuals displayed reduced capacities of cell proliferation and migration, altered myofibroblastic differentiation, and diminished collagen remodeling, indicating that the capability of gingival repair is retarded with age. Deficiencies in gingival wound-healing were additionally confirmed with a rat model [38]. Interestingly, probably for compensating reduced proliferative capacity, gingival tissues from aged animals showed higher anti-apoptotic and lower pro-apoptotic gene expression compared with gingival tissues from young animals [39].

## 3. Anatomical and Physiological Homeostasis in the Periodontal Ligament

The periodontal ligament provides the supporting connection of the cementum covering the tooth root to the inner socket of the alveolar bone through bundles of type I collagen named Sharpey’s fibers [40]. The periodontal ligament also helps to fuel vascular supply and nutrients to the cementum and the alveolar bone. The periodontal ligament consists of numerous cell types including fibroblasts, progenitor cells, epithelial cells, blood cells [23]. In addition, cementoblasts are lined at the cementum interface, while bone-associated cells, such as osteoblasts, osteoclasts, and odontoblasts, are located along the alveolar bone. Intercellularly, poroelastic substances composed of an organic matrix filled with a fluid component inside the periodontal ligament sense applied physical forces in the bone and act as an elastic limit to prevent tooth damage [41,42].

In the periodontal ligament, fibroblasts are the predominant cell type, making up approximately 25–30% of the volume space and 50–60% of the total cellularity [43]. Fibroblasts perform central roles in repair and remodeling of the periodontal ligament by generating the collagen fibers. Given that fibroblasts have the capacity to proliferate rapidly and migrate dynamically, these cell populations show a critical function during periodontal wound healing. Interestingly, when fibroblasts are exposed to physical or biochemical stimuli, they are able to differentiate into osteoblasts and/or cementoblasts by inducing c-fos mRNA expression and/or activating the ERK/JNK pathway [44,45]. The periodontal ligament fibroblasts can also be involved in osteoclastogenesis [46], suggesting the prominent role of the periodontal ligament in periodontium homeostasis.

Defense cells, including macrophages, mast cells, and eosinophils, also exist in the periodontal ligament. The primary role of these cells is the protection of periodontium by phagocytizing invading pathogens and dead cells. However, they also mediate the proliferation of fibroblasts and endothelial cells by secreting growth factors and cytokines. Another important cell type residing in the periodontal ligament is stem cell. The existence of a putative stem-cell population inside the periodontal ligament was first identified in 2004 [47]. The periodontal ligament stem cells represented mesenchymal stem cell traits such as clonogenicity, expression of specific markers STRO-1 and CD146/MUC18, and the capacity of differentiation into cementoblast, osteoblast, adipocyte, odontoblast, and fibroblast. Recent evidence additionally identified that epithelial stem cells also exist in the periodontal ligament [48].

A modest to severe functional reduction in the periodontal ligament accompanies aging. Earlier observation indicated that aging negatively influenced cell and fiber density, organic matrix production, and cellular mitotic activity in the periodontal ligament [49]. In line with these results, Benatti et al. showed that cell proliferation, mineral nodule formation, and mRNA expressions of type I and III collagen were downregulated over the lifespan [50]. The decline of proliferative capacity by aging ultimately leads to impaired wound healing and reduced tissue regeneration. Furthermore, senescent cell populations are progressively accumulated and the activity of AP-1 transcription factor is gradually decreased with age, due to a loss of c-fos expression [51]. On the other hand, mRNA levels of matrix metalloproteinase (MMP)-2, MMP-8, and tissue inhibitor matrix metalloproteinase (TIMP)-1 were upregulated, suggesting that extracellular matrix might be easily degraded with age. 

Aged periodontal ligament cells express higher level of pro-inflammatory genes including osteoprotegerin, IL-1β, and IL-6 [52]. Considering that osteoclast activity is regulated by osteoprotegerin and RANKL [53], it is not surprising that bone turnover is increased with age [54]. Aged periodontal ligament cells also show lower alkaline phosphatase activity, which means reduced osteogenesis and calcification [55]. Consistently, osteoblastic gene expression gradually diminishes with age [56]. 

Lim et al. also showed that the width of the periodontal ligament space is reduced over the lifespan [54]. This phenomenon can be explained by the accumulation of mineralized tissue and/or atrophy of collagen fibers with age. Narrower diameter of the periodontal ligament can increase the risk of tooth or bone fracture because occlusal forces will be converged on a smaller area.

## 4. Molecular Profile Changes with Age in the Periodontium

### 4.1. Inducible Nitric Oxide Synthase

According to the oxidative stress theory of aging, accumulation of oxidative damage by reactive oxygen and nitrogen species (RONS) contributes to the process of normal aging as well as the development of pathological conditions [57]. At a molecular level, excessive levels of exogenous or endogenous RONS result in oxidation of intracellular components, such as DNA, RNA, protein, and lipid [58]. Oxidative modification to these macromolecules causes disturbance of normal cellular physiological activities and impairment of homeostatic maintenance including mitochondrial integrity and proteostasis, which can ultimately lead to cell dysfunction or even cell death [59].

Nitric oxide (NO), a diatomic free radical and weak oxidant [60], is generated from L-arginine by three major isoforms of nitric oxide synthase (NOS): endothelial NOS (eNOS), neuronal NOS (nNOS), and inducible NOS (iNOS) [57]. Physiological levels of NO show prominent cellular functions via homeostatic redox-sensitive signaling pathways [61]. However, uncontrolled production of NO can participate in a variety of disease processes. When NO reacts with the superoxide anion radical (O^2−^), peroxynitrite (ONOO^-^) is actively generated and peroxynitrite mediates the nitration of target proteins at tyrosine residue. Peroxynitrite and protein tyrosine nitration are associated to the normal aging process and onset of various diseases including atherosclerosis [62], inflammatory injury [63], and neurodegeneration [64].

While the constitutive NOS isoforms, eNOS and nNOS, generate short-lasting and low levels of NO, iNOS produces long-lasting and larger quantities of NO [65]. In this reason, the contribution of iNOS to normal aging and pathological process in the periodontium has been studied. Regardless of exogenous stimuli, gingival fibroblasts obtained from aged human secreted more NO than cells derived from younger human. Consistently, the basal mRNA level of iNOS was upregulated in aged gingival fibroblasts [66].

### 4.2. Matrix Metalloproteinase (MMP)

Extracellular matrix (ECM) is a non-cellular structure that provides a biochemical and biomechanical environment within which cells reside. In addition to providing supportive structure for cells, the ECM defines the boundary between tissues and regulates cellular physiologies and intra-/inter-cellular communication. Aging affects the composition and structural flexibility of ECM. Recently, Tanaka et al. analyzed plasma proteins of all age groups and identified ~650 age-associated proteins in which extracellular matrix-related proteins were enriched [67]. Another recent study demonstrated that abundance of hyaluronan, an ECM glycosaminoglycan, gradually decreased with age, leading to the alteration in biomechanical properties of ECM [68]. As a critical factor for skin moisture, hyaluronan preserves the hydration of skin, thereby suppresses skin aging. Hyaluronan is also involved in anti-inflammatory responses and anti-photoaging of skin [69]. In addition, hyaluronan has protective functions by inhibiting parasites invasion and tumor metastasis [70]. Furthermore, hyaluronan promotes wound healing, and the prolonged upregulation of hyaluronan is associated with scar-free repair [71]. Based on these positive roles, hyaluronan is now actively used in the dental field [72]. Therefore, aging accompanying with progressive decline of hyaluronan severely impairs proliferating and regenerative capacities of oral fibroblasts, resulting in downregulation of ECM production, disorganization of ECM architecture, and inefficient wound healing [73,74]. Indeed, accumulating clinical evidence reveals that wound healing capacity after biopsy or periodontal surgery is higher in younger individuals [75,76].

**Figure 2:**
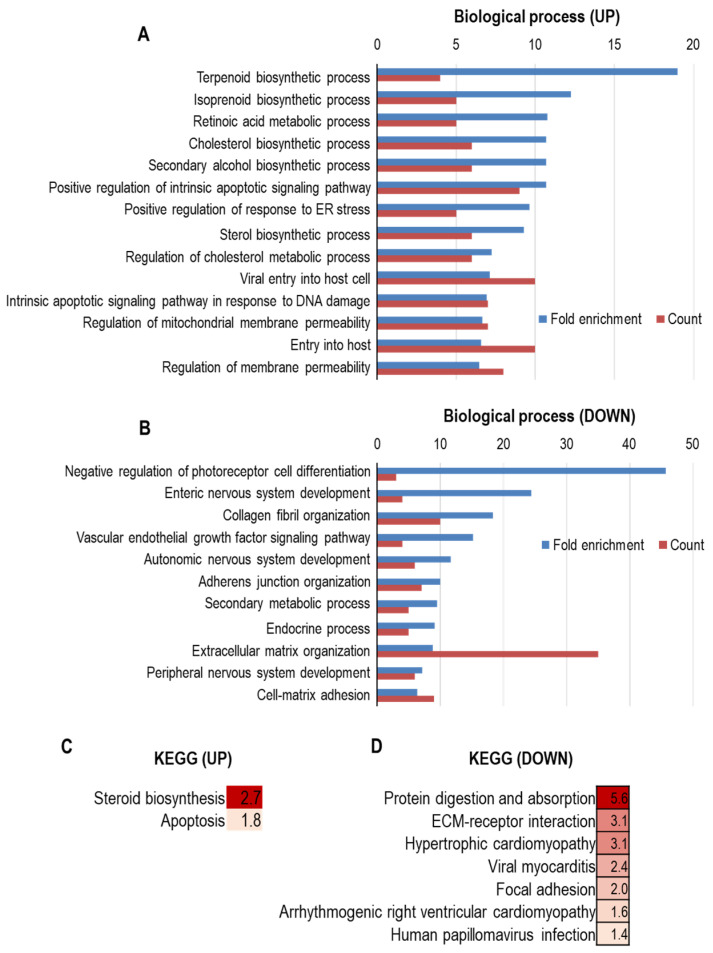
GO enrichment analysis and KEGG pathway enrichment analysis of differentially expressed genes by aging in human gingiva. (**A**–**D**) mRNA profiles of young and old human gingiva were extracted and analyzed from the NCBI Gene Expression Omnibus (GEO) profile database (GEO accession: GSE83382). Determination of upregulated genes during aging was based on fold-change of the expression level > 2 and a *p*-value < 0.05. Determination of downregulated genes was based on fold-change of the expression level < 0.5 and a *p*-value < 0.05. GO analysis of upregulated genes (**A**) and downregulated genes (**B**) was conducted by utilizing Gene Ontology resources [78,79,80]. KEGG pathway analysis of upregulated genes (**C**) and downregulated genes (**D**) was conducted by utilizing g:Profiler [81,82]. In (**C**,**D**), the color indicates the degree of statistical significance, and negative log10 of adjusted p values were presented in each box.

MMPs are Zn^2+^- and Ca^2+^-dependent proteases that have critical roles in diverse biological and pathological processes. In human, 23 different MMPs have been identified so far. Given that MMPs facilitate the disassembly of the ECM, they serve important roles in ECM homeostasis and remodeling. Notably, Kim et al. recently reported that alterations in MMPs expression would be highly responsible for gingival aging [77]. This study identified that mRNA levels of MMP-3, MMP-9, MMP-12, and MMP-13 were dramatically increased with age in gingival tissue, along with the upregulation of IL-1β expression. More interestingly, increased levels of MMPs and IL-1β would be linked to induced susceptibility to pathogenic infection in aged gingiva (Figure 2).

**Figure 3:**
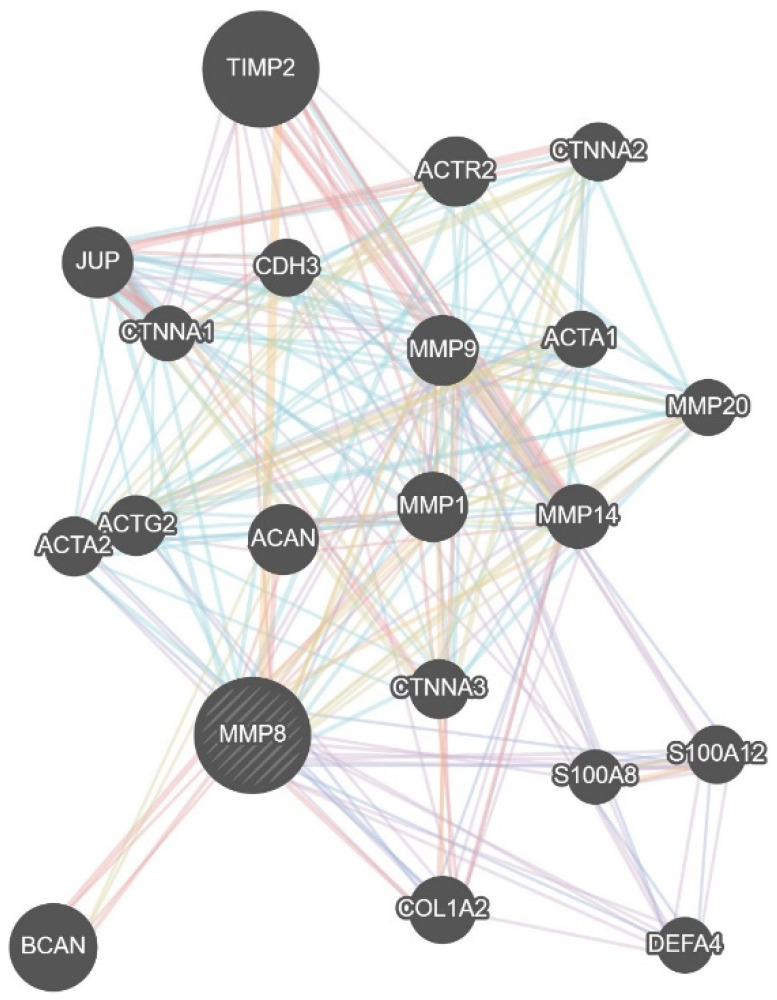
Biological network of MMP8 gene. The gene–gene interaction network of MMP8. The data were derived from the GeneMANIA database (https://genemania.org/) [94].

Considering that gingival tissues-derived MMP-3 contributes to the progression of adult periodontitis by activating MMP-8 and MMP-9 derived from crevicular fluid neutrophil [83], increased expression of MMPs would be responsible for age-related periodontal inflammation. Indeed, accumulating evidence has suggested the link between MMPs and periodontitis. MMP-8 and MMP-9 have been frequently found to be elevated in chronic or advanced periodontitis, assessing the potential of these MMPs as the most promising periodontitis biomarkers (Figure 3) [84,85]. Although expressed at a lesser degree, MMP-13 has also involved in destructive periodontal disease [86,87]. These MMPs induce the secretion of pro-inflammatory cytokines, including TNFα, IL-1β, IL-6, and IL-12, in the periodontal tissues during periodontitis progression [88]. It has been repeatedly shown that local and systemic inflammatory responses were downregulated in MMP-8-deficient mice [89,90,91]. In addition, polymorphisms in MMP-1, MMP-3, MMP-8, and MMP-9 are associated with chronic periodontitis susceptibility [92,93].

### 4.3. Others

Many research groups keep identifying the change in molecular expression during aging. Grzibovskis et al. showed that expression level of basic fibroblast growth factor (bFGF) was significantly decreased with age [95]. As a well-known growth factor, bFGF regulates the proliferation, apoptosis, and regeneration of several cells/tissues, including gingiva and periodontal ligament [96,97]. More recently, it was reported that bFGF inhibits periodontal inflammation by suppressing CD40-mediated inflammatory signaling [98]. Based on the regenerative capacity of bFGF, many clinical trials were already performed to recuperate periodontal tissue which was disrupted by age or pathological conditions. In the phase II clinical trial, administration of recombinant human (rh)-bFGF clearly increased alveolar bone height [99]. In the following clinical trials, rh-bFGF significantly enhanced bone fill, but the increment of clinical attachment level was insufficient [100,101]. When rh-bFGF was treated with a β-tricalcium phosphate (β-TCP) scaffold carrier, clinical outcomes were improved, which were determined by the clinical attachment level and linear bone growth [102].

Several genes involved in the apoptotic pathway are differentially expressed with age. González et al. showed that young gingival tissue expresses pro-apoptotic genes more abundantly than adult or aged gingiva [39]. Specifically, TNF receptor 1, BH3 interacting domain death agonist (BID), the apoptotic peptidase activating factor 1 (APAF-1), and p53 are upregulated in younger gingiva. In contrast, anti-apoptotic genes, including phosphatidylinositol 3-kinase (PI3K) and IκB kinase (IKK), were expressed higher in adult or aged gingiva. These changes in molecular profile indicate that apoptotic processes show critical function in the homeostasis of the periodontium. Earlier evidence already demonstrated that adequate apoptotic signaling produces anti-inflammatory responses, and apoptosis impairment contributed to the development of chronic inflammatory diseases [103,104]. Consistently, it was shown that upregulated resistance to apoptosis, mediated by activation of the PI3K pathway, provided survival benefit to *Porphyromonas gingivalis*-infected oral epithelial cells, resulting in periodontitis pathogenesis [105]. The molecular link between apoptosis and aging needs to be precisely determined in further studies.

## 5. Physiological Changes with Age in the Periodontium

### 5.1. Responsiveness to Pathogens with Age

Aging may directly or indirectly impact the responsiveness of host defenses against pathogens. Intrinsic aging of the skin and oral mucosa reduces the defense system to allow pathogens an easier entrance and colonization. As a result, aging promotes bacterial proliferation in the mouth, leading to the overgrowth of oral anaerobes [106]. In addition, aging influences the oral flora, showing that aged mice have less bacterial diversity when compared with young mice [107].

**Figure 4:**
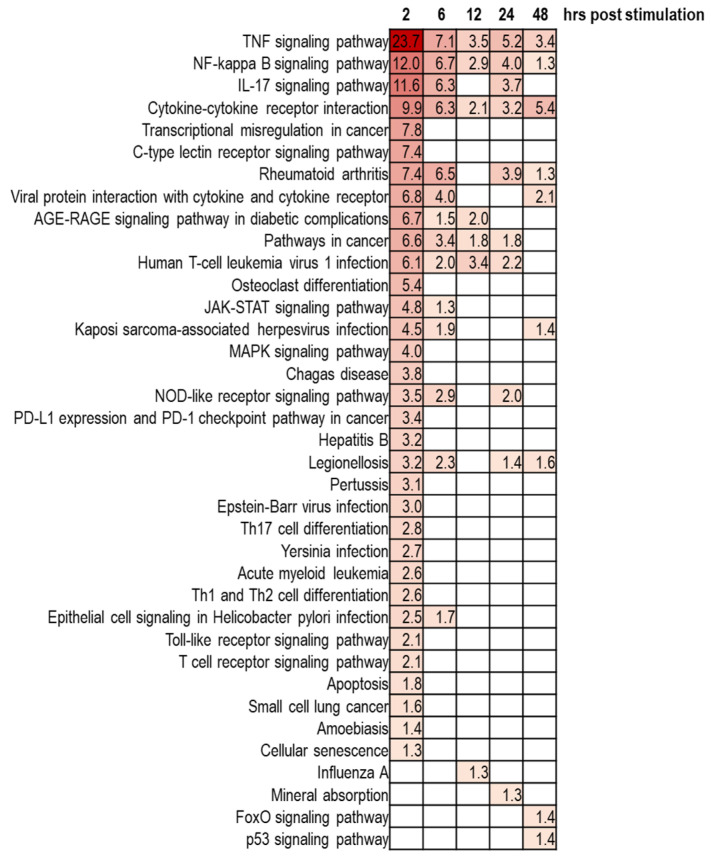
KEGG pathway enrichment analysis of upregulated genes by *Fusobacterium nucleatum* infection in human gingival fibroblasts. Gingival fibroblasts were infected with *Fusobacterium nucleatum* for 2, 6, 12, 24, and 48 h, and KEGG pathway analysis was performed. The color indicates the degree of statistical significance, and negative log10 of adjusted p values were presented in each box. Data were extracted and analyzed from the NCBI GEO profile database (GEO accession: GSE118691) [111]. Determination of upregulated genes at each time point was based on fold-change of the expression level > 2 and a *p*-value < 0.05.

The susceptibility to destructive diseases occurred in the periodontium can be affected by aging. Healthy gingival fibroblasts normally serve as the first-line guardian against oral pathogens. However, aged gingival fibroblasts show increased susceptibility to bacterial infection. When exposed to *Porphyromonas gingivalis*, the gene expression pattern revealed large discrepancy between young and aged gingival fibroblasts [108]. Particularly, aged fibroblasts failed to upregulate IL-6 production against bacterial infection, mirroring the impairment of immune responses in old fibroblasts. Ahn et al. utilized another periodontal pathogen, *Fusobacterium nucleatum*, to compare transcriptome of senescent gingival fibroblasts with that of younger cells [109], and identified that five genes (ID1, KLF10, GADD45b, TM4SF1, and CSRNP1) were mostly induced in aged gingival fibroblasts, in response to *Fusobacterium nucleatum* (Figure 4). Following study would be required to determine the roles of these genes in age-dependent susceptibility to pathogens. Moreover, further detection of new oral pathogens and investigation of their roles in the onset and progression of age-related diseases are also required [110].

Although whether saliva production and salivary flow rate are affected by age is still controversial [112,113], it has been repeatedly shown that morphologies of salivary glands change with age and atrophy of the acinar cells tends to be increased during aging [114,115]. Given that reduced salivary flow or xerostomia has been reported as side effects in more than 400 medications, including psychotropic analgesics, diuretic drugs, calcium antagonists, and anti-histamines, elderly people seem to be more afflicted with dry mouth symptoms. Considering the preventive roles of saliva, such as suppressing bacterial propagation and neutralizing acids released from pathogens, tendency of drug dependence in the elderly would contribute to increased susceptibility to oral pathogens in aged people [116].

### 5.2. Drug-Influenced Alterations in the Periodontium

Older people are more likely to be taking multiple medications to restore organ homeostasis and/or treat pathological conditions. Several medications may function as positive or negative factors on the periodontium.

Overall prevalence of hypertension increases with age consistently in all world regions [117]. Calcium channel blockers (CCBs) are drugs frequently used to reduce blood pressure. By binding to L-type calcium channels expressed on vascular smooth muscles and cardiac myocytes, CCBs lead to relaxation of vascular smooth muscles and vasodilation, which in turn lowers arterial blood pressure [118]. Interestingly, the majority of CCBs, including nifedipine, diltiazem, verapamil, and felodipine, have been reported to induce gingival enlargement or overgrowth, and gingival hyperplasia could be localized or shown in the entire mouth [119,120,121,122]. Although CCBs do not seem to directly affect the underlying alveolar bone, drug-induced gingival overgrowth may create pockets for the accumulation of pathogenic bacterial biofilm, thus inducing periodontitis and tooth loss.

In contrast, some medications used to treat other systemic diseases could provide benefits for abnormalities in the periodontium. Rheumatoid arthritis (RA) is a chronic auto-inflammatory disease, resulting in synovial hyperplasia and extensive joint destruction. Although RA can arise in any age, the cumulative risk of RA increases rapidly around age 60 years, where the incidence rate of RA reaches a zenith, and flattens beyond age 80 years [123]. Rituximab is a chimeric mouse/human monoclonal antibody against the protein CD20 that is primarily expressed on the surface of B cells. Although the role of B cells in the pathogenesis of RA is not precisely established, studies have shown that the administration of rituximab, alone or in combination with either cyclophosphamide or methotrexate, depleted circulating B cells and improved disease symptoms of RA for up to 1 year [124,125]. Coat et al. demonstrated that rituximab treatment significantly ameliorated periodontal indices, such as modified gingival index, papillary bleeding index, pocket depth and attachment loss, and delayed inflammation and bone damage [126]. Although this study did not compare the efficacy of rituximab between the young and aged group, considering that mean age of subjects in the study was over 60 years, it would be possible that rituximab may have a positive role in age-mediated pathophysiology in the periodontium.

Another example of a drug that shows potential benefits for the periodontium is metformin. Although metformin has been used as the first line pharmacotherapy against type 2 diabetes since the 1950s, metformin also showed other virtues such as AMPK-dependent anti-tumor activity [127] and anti-atherogenic functions [128]. Interestingly, metformin improved clinical outcomes in chronic periodontitis patients [129]. Metformin suppressed *Porphyromonas gingivalis* LPS-induced production of pro-inflammatory cytokines, including IL-1β, IL-6, and TNF-α, in human gingival fibroblasts and periodontal ligament cells through targeting NLRP3 inflammasome [130,131]. More recently, Kuang et al. demonstrated that metformin can prevent oxidative stress-induced cellular senescence by stimulating autophagy [132]. In addition, metformin positively regulates bone repair and metabolism by reversing abnormal expression of RANKL, osteopontin, TRAP expression [133]. Furthermore, metformin shows osteogenic activity through facilitating the proliferation and differentiation of periodontal ligament stem cells [134,135].

## 6. Conclusions

The periodontium serves multiple functions in maintaining the structure of the orofacial complex, providing mastication control, and protecting the oral cavity against pathogens. Because the destruction of the periodontium is currently considered as irreversible and permanent, structural and functional maintenance of the periodontium is critical. However, these physiological roles are progressively reduced or lost with age, which leads to the increased vulnerability to numerous diseases. Aging-related intrinsic factors, including replicative stress, oxidative damages, genetic alteration, mitochondrial decline, and cellular senescence, and extrinsic factors, including changes in oral flora and medication-driven pathologies, synergistically weaken the performance of the periodontium. Therefore, understanding the effect of aging on homeostasis in the periodontium would be important to keep the oral environment and further life quality in a good condition.

## Data Availability

Agree with MDPI research data policies.
